# Food Bolus Properties in Relation to Dentate and Prosthetic Status

**DOI:** 10.3390/healthcare10091596

**Published:** 2022-08-23

**Authors:** Elena Preoteasa, Alexandra Melania Oncescu Moraru, Daniela Meghea, Catalina Murariu Magureanu, Cristina Teodora Preoteasa

**Affiliations:** 1 Department of Prosthodontics, “Carol Davila” University of Medicine and Pharmacy, 032799 Bucharest, Romania; 2 Private Practice, 011497 Bucharest, Romania; 3 Department of Scientific Research Methodology-Ergonomics, “Carol Davila” University of Medicine and Pharmacy, 050037 Bucharest, Romania

**Keywords:** mastication, masticatory, food comminution, chewing, sieve method, dental prosthesis

## Abstract

The objective of this study is to evaluate food bolus properties (mass, moisture content and food comminution) in patients wearing fixed or removable dental prostheses. Methods: A cross-sectional study was conducted on a convenience sample of patients aged at least 55 years old. Patients chewed a 10 g sample of fresh raw carrot until they felt ready to swallow. The mass of the food bolus was determined as collected and after drying. Food comminution was assessed by the multiple sieve method. Results: Patients with fixed prostheses compared to those with removable prostheses registered a similar mass of food bolus as collected (4.40 g vs. 4.60 g; *p* = 0.856); a higher mass of dried food bolus (3.46 g vs. 0.86 g; *p* < 0.001); lower moister of food bolus (24.65% vs. 82.35%; *p* < 0.001); and better food comminution (mass of smaller particles, of size below 2 mm, represented 65.93% vs. 20% of dried food bolus). In removable denture wearers, food comminution was slightly better in partially than in completely edentulous patients, and rather similar in completely edentulous patients with either implant overdenture or complete denture in the mandible, and complete denture in the maxilla. Conclusions: The current results suggest that food bolus properties are dependent on the dentate and prosthetic status.

## 1. Introduction

During mastication, the first step of digestion initiation, solid foods are ground into smaller particles and lubricated with saliva to form food bolus [1]. Impaired oral health has been linked to mastication and nutritional problems, with a negative impact on systemic health and quality of life [2]. Concerns are raised especially in the elderly, presenting rather frequently as multiple severe oral alterations and systemic comorbidities, all by various mechanisms negatively influencing the masticatory function [3,4]. Among the oral conditions encountered in the elderly, edentulism is one with a great negative impact on masticatory performance. This presents great variability in type (e.g., complete or partial) and prosthetic rehabilitation (e.g., complete denture, implant overdentures, partial dentures, fixed tooth or implant supported prosthesis), with the latter probably associating different masticatory performance too [5]. This motivated our research, which started from the desire to find out more about the relationship between the masticatory performance and the dentate and prosthetic status. 

State-of-the-art research into masticatory performance highlights the importance of both subjective and objective methods of evaluation, as it is able to produce complementary data, frequently showing a weak association [6,7]. Moreover, objective assessments are considerably fewer than subjective ones, posing greater methodological challenges and difficulties [8]. Considering the importance of the objective knowledge of medical phenomena in general, masticatory performance included, we have chosen a method of assessment based on sieving the comminuted food, which is one of the most used in this regard [7].

Analyzing the state-of-the-art research in this area, in our opinion, one issue that is insufficiently known but of great clinical relevance is that of masticatory performance, assessed objectively in different prosthetic alternatives in the elderly. Firstly, few studies have been undertaken on this topic, and those that are available sometimes report conflicting results. Secondly, there are numerous combinations when considering the type of edentulism and prostheses in the mandible and in the maxilla; not all of them have been analyzed, as it is possible to associate different masticatory performance. Moreover, considering that oral, systemic and general factors may act as confounders, multiple studies are recommended in order to draw a conclusion with an adequate degree of certainty on the masticatory performance associated with different types of prosthetic rehabilitation. To our knowledge, a comparison between the specific prostheses analyzed in this research has not been made [8,9,10], and overall, more research is required on this topic, which is very important from a clinical perspective.

Hence, the objective of this study is to evaluate food bolus properties (mass, moisture content and food comminution) in patients wearing removable prostheses, as compared to patients wearing fixed prostheses. A secondary objective intends to compare the removable prosthesis most frequently encountered in this study sample, i.e., complete dentures in both jaws, overdenture on implants in the mandible and complete denture in the maxilla, and removable partial dentures in both jaws.

## 2. Materials and Methods

This study was approved by the Ethics Committee of “Carol Davila” University of Medicine and Pharmacy, Bucharest (PO-35-F-03 No. 6121).

### 2.1. Study Design and Participants

A cross-sectional study was conducted on a convenience sample of patients who were included on a voluntary base from the Clinic of Dental Prosthetics from Carol Davila University and Pharmacy in Bucharest. The participants submitted a written informed consent form after agreeing to participate in this study. From a dental prosthetic point of view, two categories of patients were included: patients with natural dentition or edentulism rehabilitated with fixed prostheses in both jaws (1st group); patients with edentulism rehabilitated with removable prostheses in both jaws, regardless of type, e.g., partial or complete dentures, and implant overdentures or tooth supported overdentures (2nd group). Only those patients that stated their general satisfaction regarding performing mastication who were aged at least 55 years old were included. The exclusion criteria were patients with dental prosthetic rehabilitations made less than 3 months ago; patients with oral pain or other oral problems that interfere with the habitual masticatory behavior; patients with unrestored intercalated edentulous spaces; and patients with systemic alterations with severe negative consequences on masticatory function (e.g., Parkinson and other diseases with motor control abnormalities).

Regarding the size of the study sample, the target was to have at least 10 participants in each group, at the moment of performing a comparison between the groups. This number was based on previous related studies [10,11,12]. Considering this, and the objectives of our study, we included at least 10 participants in the 1st group, and consequently, we added participants in the 2nd group until there were at least 3 subgroups consisting of at least 10 persons with the same type of prosthesis in the maxilla and in the mandible.

### 2.2. Study Variables and Data Collection

After the patients’ inclusion, interviews and clinical examinations were performed in order to confirm eligibility and collect data on general characteristics, satisfaction with masticatory performance, and on dental and prosthetic status. Data on masticatory efficiency were collected afterward, it being assessed by the food bolus assessment, i.e., mass of food bolus as collected before swallowing, mass of dried food bolus, moisture of food bolus, and food comminution assessed by particle size distribution in dried food bolus.

All the patients were asked to chew a 10 g sample of fresh raw carrot, and to expectorate it when they felt that swallowing was about to be triggered. The mass of the food bolus was registered as collected and after it was dried. The device used for determining the mass and drying of the food bolus was MB45 Moisture Analyzer (OHAUS Corporation, Parsippany, NJ, USA), which operates on the thermogravimetric principle. At the start of the measurement, the mass of the sample was determined with this device and recorded. Afterwards, the sample was heated by the integral halogen dryer unit and moisture was vaporized. The temperature used was 85 °C. On completion of drying, the mass of the sample was displayed and recorded. The duration of the process was 10 min, of which 3 min was for preheating, 5 min was for drying and 2 min was for the cooling phase. In addition to the chewed samples, a sample of fresh raw carrot that was not chewed was dried and its mass was determined as being of 6.28 g. 

The moisture of the food bolus was calculated based on mass loss after drying. The formula used was the following: moisture of food bolus = (food bolus mass loss after drying/mass of food bolus as collected) × 100.

Food comminution was assessed by the particle size distribution of the dried food bolus, by the multiple sieve method, using Vibratory Sieve Shaker ANALYSETTE 3 PRO (Fritsch GmbH, Idar-Oberstein, Germany). The dried food bolus was passed through a series of sieves of progressively smaller mesh size i.e., >4 mm; between 2 mm and 4 mm; between 1 mm and 2 mm; between 0.7 mm and 1 mm; and <0.7 mm. The mass of the material that was stopped by each sieve was displayed. In order to determine if there were differences in food comminution between groups, the masses of the particle of the five different size categories that resulted from sieving were compared independently. Masticatory performance was considered better if chewing resulted in more small-sized particles, or in other words, if the mass of the smaller particles was higher. Moreover, the general pattern of the particle size distribution in the patients with different prostheses was graphically displayed and analyzed. 

**Figure 1:**
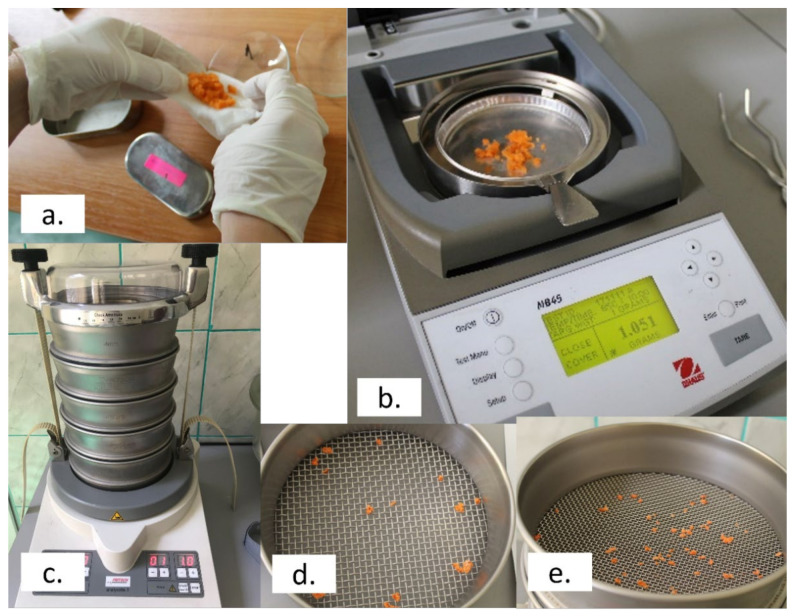
Workflow with main operational steps: (**a**) food bolus collected just before swallowing; (**b**) determining the mass and drying of the food bolus (device used: MB45 Moisture Analyzer; OHAUS); (**c**–**e**) assesssing food comminution by multiple sieve method (device used: Vibratory Sieve Shaker ANALYSETTE 3 PRO; FRITSCH).

Workflow is shown in Figure 1.

### 2.3. Data Analysis

Comparisons were made first between the patients with fixed and removable prostheses. Secondly, the most frequently encountered removable prosthetic rehabilitations in the study sample were compared, i.e., removable partial denture in both jaws; implant overdenture in the mandible and complete denture in the maxilla; complete dentures in both jaws. 

SPSS Statistics was used for the data analysis. As the data were not normally distributed, nonparametric tests were used. Chi-square, Mann–Whitney U and Kruskal–Wallis tests were used for group comparison. Following a significant Kruskal–Wallis test, the Dunn post hoc method from SPSS Statistics was used. The statistical significance level was set at *p* < 0.05.

## 3. Results

The study sample included 106 patients, of which 64 were females and 42 males, with a median age of 69 years old (age ranging from 57 to 84 years old). Eleven patients had edentulism rehabilitated with fixed prostheses in both jaws, and 95 patients had edentulism rehabilitated with removable prostheses in both jaws. Sex was not statistically significantly different between the groups (six males rehabilitated with fixed prostheses in both jaws, 36 males rehabilitated with removable prostheses in both jaws; *p* = 0.285). The patients with removable prostheses in both jaws were statistically significantly older than the patients with fixed prostheses in both jaws (median 69 years old vs. 61 years old; *p* < 0.001). This age difference was considered as acceptable for group comparison in regard to the masticatory performance, considering that the values of the medians were in the same age decade, and also considering the research of Kim et al. [13], which offers evidence that the persons with at least 26 remaining teeth, fixed dental prostheses included, show a similar masticatory performance regardless of their age group. The most frequently encountered removable prosthetic rehabilitations in the study sample were complete dentures in both jaws; overdenture on implants in the mandible and complete denture in the maxilla; and removable partial dentures in both jaws (Table 1). The patients with these three types of removable prostheses were similar as regards sex (*p* = 0.927) and age (*p* = 0.069). 

### 3.1. Mass of Food Bolus as Collected and after Drying

The patients with fixed prostheses in both jaws compared to those with removable prostheses in both jaws registered a similar mass of food bolus as collected before swallowing, but a statistically significant higher mass of dried food bolus and a lower moister content of food bolus (Table 2).

The mass and moisture of the food bolus collected from the patients with removable prosthetic rehabilitations that were most frequently encountered in this study sample were compared (Table 3). The mass of food bolus as collected before swallowing registered similar values regardless of the patients’ prosthetic status, the difference not being statistically significant. The mass of food bolus after drying and moisture content were statistically significantly different according to prosthetic status. A higher mass of food bolus after drying and a lower moisture of food bolus were observed in the partially edentulous patients (restored with removable partial dentures in both jaws) than in the completely edentulous patients (restored either with complete denture in both jaws, or with implant overdenture in the mandible and complete denture in the maxilla). Pairwise comparisons showed a statistically significant difference only between the patients with removable partial dentures, and implant overdenture in the mandible and complete denture in the maxilla (*p* = 0.004 for food bolus mass after drying; *p* = 0.009 for moisture of food bolus).

### 3.2. Food Comminution

**Figure 2:**
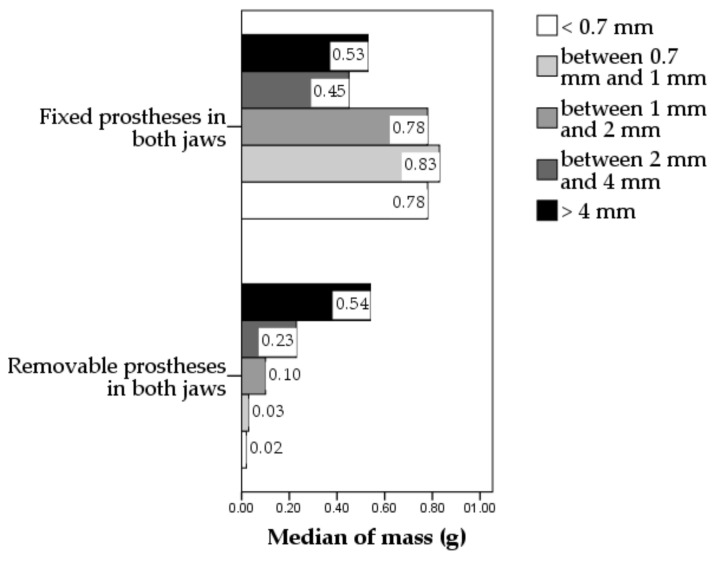
Particle size distribution in patients wearing fixed or removable prostheses.

The dried food bolus contained food particles of different sizes. The particle size distribution in the bolus was considerably different in the patients with fixed prostheses in both jaws compared to the patients with removable prostheses in both jaws (Figure 2). The mass of the biggest particles (i.e., above 4 mm) was similar in these two groups (*p* = 0.682), but the mass of the particles of lower sizes was statistically significantly higher in the group of patients with fixed prostheses in both jaws compared to the patients with removable prostheses in both jaws (*p* < 0.001 for all four categories of particles with sizes below 4 mm). In the patients with removable prostheses in both jaws, as a general pattern of particle size distribution, there was a decrease in the mass of the particles from the biggest to the lowest size category, mass of smaller particles, of size below 2 mm, having a median of only 20% of the mass of dried food bolus. In the patients with fixed prostheses, the particle size distribution was very different compared to the previous, the majority of the mass being composed of particles sized below 2 mm, their mass having a median of 65.93% of the mass of dried food bolus. This indicated that food comminution was better in the patients with fixed prostheses than in those with removable prostheses.

**Figure 3:**
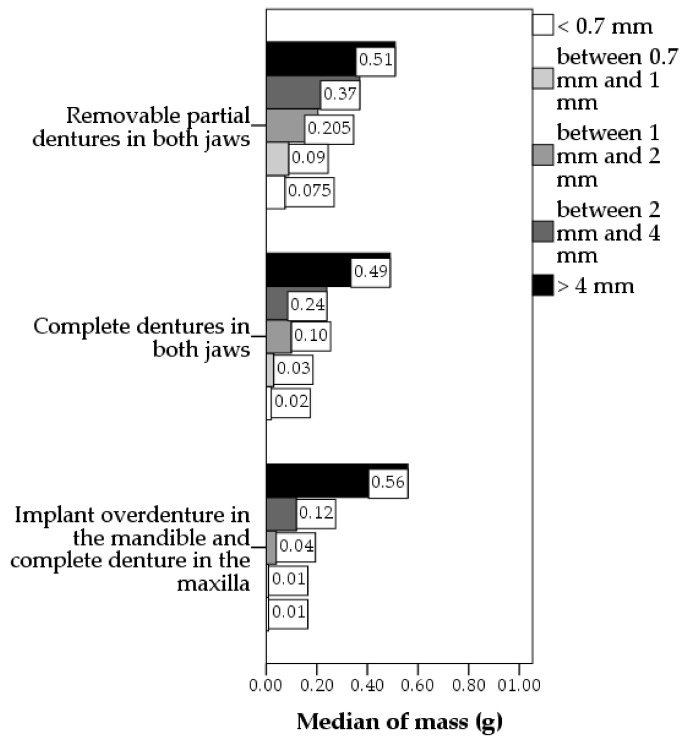
Particle size distribution in patients with removable prostheses rehabilitations that were most frequently encountered in this sample.

Particle size distribution was compared among the patients with removable prosthetic rehabilitations that were most frequently encountered in this study sample (Figure 3). The general pattern for all types of removable prosthetic rehabilitations was similar in regard to a noticeable decrease in the mass of the particles from the biggest to the lowest size category. The mass of the biggest particles (i.e., above 4 mm) was similar in these groups (*p* = 0.886), but the mass of the smaller size particles was statistically significantly different (*p* < 0.001 for all four categories of particles with sizes below 4 mm). The mass of particles below 2 mm was the highest in patients with removable partial denture in both jaws (median 25.43%), intermediate in patients with complete denture in both jaws (median 21.74%), and the lowest in patients with implant overdenture in the mandible and complete denture in the maxilla (median 10.87%).

## 4. Discussion

This study’s results indicate that food bolus properties such as mass, moisture and food comminution are different between patients with fixed or removable prostheses. The mastication efficiency in terms of food comminution is considerably better in patients with fixed prostheses in both jaws than in patients with removable prostheses in both jaws. Moreover, it is suggested that even if food fragmentation has great similarities between patients with removable prostheses, it is slightly better in removable partial dentures wearers, compared to completely edentulous patients with either implant overdenture or complete denture in the mandible and complete denture in the maxilla.

The mass of food bolus registered differences between the patients with different prosthetic status. The mass of food bolus as collected when the patients felt ready to swallow was similar in all patients, but the mass of food bolus after drying was considerably higher in the patients with fixed prostheses in both jaws compared to those with removable prostheses in both jaws, mass loss after drying being considerably lower in the first. The food bolus is a mixture of food and saliva, and according to this study’s results, their proportion is highly variable according to the dentate and prosthetic status. The higher moisture content of food bolus in patients with removable dental prostheses may be seen as a biological response to the mastication deficiencies. The decreased fragmentation of solid food may be compensated by an increase in the moisture content of the food bolus. These results suggest that the outcome of mastication in terms of food bolus being perceived as ready for swallowing is a food bolus of similar mass, regardless of the prosthetic status, with big differences in moisture content and food fragmentation. These results are in accordance with the study of Peyron et al. [14] who found that after a complete masticatory cycle the weight of the food bolus was about 40% of the initial food sample, similar for carrot and other food samples. This low retrieval rate of the initial food sample may be explained by various factors, such as intermediate swallowing and the liberation of the liquid content of food [14,15]. Another aspect to be considered is the decrease in food solid mass after drying, which was seen in all patients regardless of their dentate and prosthetic status (the mass of sample dried without being chewed was 6.28 g). This was found at a greater extent in removable prostheses wearers and might be explained as a greater retention of pieces of solid food under the removable prostheses, at a greater risk in this regard being the smaller particles, which are also the ones that were considerably different in this study between the patients wearing fixed or removable prostheses.

Food comminution was considerably different between the patients wearing fixed or removable prostheses. As a general pattern observed in this study, better food comminution resulting in a higher proportion of particles of smaller size is dependent on the dentate and prosthetic status. Food comminution was the best in patients with fixed prostheses, and the lowest in complete edentulous patients rehabilitated by complete denture or implant overdenture in the mandible and complete denture in the maxilla. These results were to be expected as tooth loss associates the loss of periodontal sensitivity with negative impact on feedback controls for the formation of food bolus. Consequently, prosthetic rehabilitations are seeming to improve, but not to the full extent, the masticatory function [11,16]. As Carlsson [17] stated, the best chance to preserve a good mastication efficiency is given by maintaining a reasonable number of natural teeth. Moreover, differences in mastication, including food comminution, are probably linked to the particularities of the prosthetic rehabilitation, e.g., occlusion scheme; tooth-, implant-, or mucosal support; and the material of artificial teeth [18]. From the latter, occlusal support was very different between the patients in this study sample, basically because masticatory efficiency in tooth-supported and mainly mucosal-supported prosthetic restorations were compared. This may be an important factor that explains the great dissimilarities between study groups, as it was previously shown that an adequate occlusal support is an important factor for maintaining efficient chewing [19].

When analyzing the relationship between particle size distribution and the insalivation of food bolus, it was observed that when comminution resulted in a higher proportion of particles with a bigger size, as in removable prostheses wearers, a better insalivation of food bolus was observed; in other words, moister content was increased, this probably being a factor that contributes to making food suitable for deglutition and digestion. Considering the previous, removable denture wearers with rather frequently altered salivary flow may exhibit even greater difficulties in chewing and present a higher risk of digestive problems in general (swallowing food particles of increased size that are insufficiently insalivated may be linked to increased gastric and intestinal motility and, respectively, to the increased amount of secreted gastric acid) [20]. In these patients, the prosthetic alternatives that favor food fragmentation in smaller pieces, which might require less insalivation of food bolus, may be more recommended.

There is a great body of evidence in the scientific literature that confirms that masticatory performance is different according to multiple factors, e.g., age, gender, systemic status, oral health, dentate and prosthetic status [11,14,15,16,17]. Even so, considering that mastication is assessed from multiple points of view and has multiple methods of being assessed (food comminution being only one aspect to consider), the results from studies are not always similar. Even so, all studies can increase knowledge on it and help in identifying ways to improve prosthetic treatments in this regard. In general, the mastication of dentate is seen to be better than the one of edentulous patients, and prosthetic restorations improve masticatory performance, but prosthetic treatment alternatives have many subcategories with differences that probably impact mastication in variable ways [21,22]. 

Studies that analyzed mastication in patients with different conventional prosthetic rehabilitation and used comminution tests in this regard found results that are in general consistent with the ones from this study. According to our results, food comminution is better in patients with fixed prostheses compared to patients with removable dentures, as confirmed by previous research [9]. Our study results also suggest that the mass of particles of bigger size, above 4 mm, is similar regardless of dentate or prosthetic status. This result can be seen as being concordant with the one of Sugimoto et al. [23], which concluded that comminution performance immediately before swallowing is not statistically different between dentate and denture-wearing patients by analyzing only bigger particles, of a size above 2 mm. According to Velástegui and Salazar [10], masticatory performance in terms of food fragmentation is better in removable partial dentures than in complete denture wearers, and the data are supported by our study results. According to our results, the pattern of comminution in removable dentures wearers has great similarity. Other studies found that there is no difference among various types of prostheses, such as the study of Bessadet et al. [11], who found that rehabilitation with removable prostheses improves food fragmentation, but a difference was not noticed between Kennedy classes. 

Studies that analyze mastication in patients with different implant prosthetic rehabilitation and used comminution tests in this regard usually conclude that their use is beneficial. According to the study of Gonçalves et al. [24], in partially edentulous patients, the rehabilitation with implant-supported fixed partial dentures compared to implant-supported removable dentures significantly reduces median particle size. The research of Sharma et al. [25] offered evidence that the replacement of mandibular conventional denture with an implant-supported mandibular overdenture considerably reduces mean particle size. Disagreeing with the previous study, but in accordance with this study’s results, the randomized controlled trial reported by Fontijn-Tekamp et al. [26] found that there is no difference between patients with complete dentures and implant overdentures regarding food comminution, and the study of Van Doorne et al. [8] showed that objective masticatory performance is similar in wearers of complete denture and mini dental implant overdentures. The lack of improvement of food fragmentation in mandibular implant overdentures compared to conventional dentures might be related to retention and stability problems, known as factors that impair mastication in bimaxillary complete denture wearers [27]. Applying dental implants only in the mandible, as in our study, may be associated with stability problems of the maxillary complete denture, thus, negatively influencing masticatory performance. Moreover, a similar chewing performance of implant overdentures and complete denture can be explained by their similar characteristics from an oral and prosthetic point of view (e.g., occlusal scheme; osseous and mucosal support; features related to complete edentulism state such as loss of periodontal sensitivity and impaired oral stereognostic ability). Usage of dental implants to increase the retention of mandibular complete denture is beneficial from many points of view, and probably has a positive effect on a patient’s satisfaction regarding the performance of mastication, but it should be better known to what extent it has an objective positive effect on masticatory performance [28].

Mastication is linked to both oral health and systemic health by various mechanisms. Depending on the systemic disease and its related treatment, aspects such as impaired salivary function, alterations of the oral mucosa, and muscular weakness may be encountered, with negative effects on masticatory performance. Furthermore, masticatory deficiency may have a negative effect on systemic health, e.g., risk factor for gastrointestinal disorders and cognitive dysfunction [29]. Taking into account the results of this study that suggest that food bolus properties are considerably different according to the type of prosthetic rehabilitation, dental treatment in the case of patients affected by systemic diseases should be conducted so a healthy masticatory function may be preserved. For example, oncological patients frequently show a higher prevalence of dental caries, fewer teeth and occlusal units, decreased salivary flow, and oral mucosal injuries, these being some of the factors that contribute to their identified worse masticatory performance [30]. In the context of the positive effect that adequate mastication has on health status and quality of life, treatment should consider aspects such as preserving as many teeth as possible, choosing dental prostheses that have the best outcome in terms of masticatory efficiency, and the prophylaxis of some commonly encountered complications of cancer treatments with negative effects in this regard, e.g., palifermin can be used for the prevention of severe oral mucositis [31]. Moreover, methods of delivering dental care, with influences sometimes on choosing between treatment options and sometimes between the prosthetic alternative, can be related to contextual factors, as recently occurred during the COVID-19 pandemic [32]. For example, during this period, especially in the beginning, less invasive procedures were preferred by both dental professionals and patients; some of the dental treatments were postponed and different anti-contagion strategies were tested and sometimes implemented, e.g., the use of more personal protective equipment by dental professionals, and air purifiers [32,33,34]. 

The evaluation of masticatory efficiency can be carried out by objective or subjective tests, both offering valuable information that is needed for a better understanding of this process, and consequently, methods of improving prosthetic rehabilitations. The multiple sieve method used in this study is an objective test considered as being more reliable comparative to others, such as the single sieve method [35]. Basically, interpretation is given on an analysis of particle sizes, referring to mastication being rated as better when a greater proportion of particles ground by mastication are of smaller sizes. Even so, this test and other comminution tests reported in the scientific literature, some of those being referred to in this article, have protocols that differ in many ways, e.g., the use of natural or artificial test food; the time of the collection of the bolus; sieves used (different as number and aperture); usage or not of the drying of the food bolus (for the drying of food bolus various methods, devices and parameters are found); particles size to be considered; and methods of data analysis [36,37]. These differences of the comminution methods and differences from other objective methods of evaluation may lead to different results and contribute to difficulties in comparing data [16,38,39]. Therefore, deeper knowledge is needed on the objectives of methods of evaluating masticatory efficiency, in order to identify those that provide better data from a clinical perspective.

Study limitations as a possible source of errors include: sample size, considering that the different groups compared were relatively small, similar to many studies that analyzed masticatory efficiency by comminution tests, this being a factor that interferes with findings’ generalizability; the cross-sectional design of the study; the use of only one food sample (i.e., fresh raw carrot) and its characteristics (carrot samples could present some hardness differences when compared to the more constant hardness of artificial test food); not including more prosthetic rehabilitation alternatives, only a few of them being considered; not including patients with intact natural dentition, this being due to the fact that persons with this particularity above 55 years old were not identified during recruitment; age difference between patients with different prosthetic status. Even so, this research provides data on this vast topic, which nowadays requires better knowledge in order to improve patients’ quality of life.

## 5. Conclusions

The current results suggest that food bolus properties are dependent on the dentate and prosthetic status. Food comminution is considerably better in patients with fixed prostheses in both jaws than in patients with removable prostheses in both jaws, and even if it displays high similarities between patients with removable prostheses, it is slightly better in removable partial dentures wearers, compared to completely edentulous patients with either implant overdenture or complete denture in the mandible and complete denture in the maxilla. Aspects such as the higher moisture content of the food bolus in patients with removable prostheses may be seen as a biological response to the comminution deficiencies. 

## Figures and Tables

**Table 1:** Study sample’s prosthetic status.

Fixed Prostheses in Both Jaws (n = 11)					
Removable Prostheses in Both Jaws (n = 95)					
		Maxilla			
		Removable Partial Denture	Tooth Supported Overdenture	Implant Overdenture	Complete Denture
Mandible	Removable partial denture	n = 12	-	-	n = 7
	Tooth-supported overdenture	n = 5	n = 7	-	n = 3
	Implant overdenture	-	n = 1	n = 7	n = 15
	Complete denture	n = 3	-	-	n = 35

**Table 2:** Median of mass and moisture of food bolus in patients wearing fixed or removable prostheses.

	Fixed Prostheses in Both Jaws (n = 11)	Removable Prostheses in Both Jaws (n = 95)	*p* ^a^
Food mass before chewing	10 g	10 g	>0.999
Food bolus mass as collected	4.40 g	4.60 g	0.856
Food bolus mass after drying	3.46 g	0.86 g	<0.001
Moisture of food bolus	24.65%	82.35%	<0.001

^a^*p*-values were calculated with Mann–Whitney test.

**Table 3:** Median of mass and moisture of food bolus collected from patients with removable prostheses rehabilitations that were most frequently encountered in this sample.

	Removable Partial Dentures in Both Jaws (n = 12)	Complete Dentures in Both Jaws (n = 35)	Implant Overdenture in the Mandible and Complete Denture in the Maxilla (n = 15)	*p* ^a^
Food mass before chewing	10 g	10 g	10 g	>0.999
Food bolus mass as collected	4.10 g	4.50 g	4.80 g	0.089
Food bolus mass after drying	1.29 g	0.86 g	0.75 g	0.004
Moisture of food bolus	64.79%	81.89%	84.81%	0.005

^a^*p*-values were calculated with Kruskal–Wallis test.

## Data Availability

The data that support the findings of this study are available from the corresponding authors, C.T.P., A.M.O.M. and C.M.M., upon reasonable request.
